# Correspondence

**Published:** 1911-08

**Authors:** Henry R. Harrower

**Affiliations:** 60 W. Randolph St.; Chicago, Ill.


### Correspondence.

CHICAGO, ILL., June 30, 1911.

*To the Editor of the Texas Medical Journal:*

With your kind indulgence I want to bring an important message to your readers. I am going to alternate my bi-monthly journal, *Physiologic Therapeutics,* with "another new journal," which will bear the attractive title "Successful Medicine." I believe that many of your readers will welcome this new journal, as it is to be a journal of commercial medicine, devoted solely to that side of practice which directly concerns the dollars and cents, published with the main idea of making its readers better paid practitioners than more bricklayers or other skilled laborers who gain their skill with comparatively little sacrifice and who earn more than the average physician.

The financial side of practice is mighty important. They talk about the "science of medicine" and the "art of medicine," but if I am not badly mistaken the dollars and cents side of medicine has been too lightly considered. Not, mind you, that the scientific and altruistic side of the doctor's work is depreciated—far from it. Medicine today, as always, is filled with honest, scientific workers, and with men ever ready to help "the under dog" when opportunity affords; but think of $700 a year as the income of the average American physician! Remember, too, that medicine unfortunately takes no note of the eight-hour law.

*Successful Medicine* will deal with the problem of the physician in his office, and especially with those which concern the business of getting results and the money for it. It will be published bimonthly, beginning in September. It will be regular magazine size with a minimum of 48 pages, and the price will be only 25 cents a year! Since the journal will be devoted to such an important phase of medicine, surely every reader of the TEXAS MEDICAL JOURNAL must be interested. How many will subscribe probably in advance of publication?

Cordially yours,

HENRY R. HARROWER, M. D.

60 W. Randolph St.

---

The surgeon who adopts the rule not to tie off the neck of an inguinal hernia sac until he has dissected from it all doubtful fatty masses will save himself the embarrassment of occasionally injuring the bladder.—*American Journal of Surgery.*

